# *BMC Immunology* reviewer acknowledgement 2014

**DOI:** 10.1186/s12865-015-0081-4

**Published:** 2015-04-21

**Authors:** Christopher Morrey

**Affiliations:** BioMed Central, Floor 6, 236 Gray′s Inn Road, London, WC1X 8HB UK

## Abstract

The editors of *BMC Immunology* would like to thank all of our reviewers who have contributed to the journal in Volume 14 (2014).

**Alok Agrawal**

USA

**Zaied Ahmed**

Japan

**Carlos Alfaro**

Spain

**Irving Allen**

USA

**Brad Amendt**

USA

**Clark Anderson**

USA

**Kazunori Aoki**

Japan

**Gamal Badr**

Egypt

**Brent Berwin**

USA

**Joseph Blattman**

USA

**Ester Boix**

Spain

**Jean-David Bouaziz**

France

**Prosper Boyaka**

USA

**Cyro Brito**

Brazil

**Franco Buonaguro**

Italy

**Marzia Caproni**

Italy

**Arturo Casadevall**

USA

**Sofia Casares**

USA

**Filippo Castiglione**

Italy

**Jean-Marc Cavaillon**

France

**Jan Ceuppens**

Belgium

**Bryce Chackerian**

USA

**Divaker Choubey**

USA

**Stephen Cobbold**

USA

**Fabrice Cognasse**

France

**Behazine Combadiere**

France

**Divi Cornec**

France

**David Cousins**

USA

**Julio Croda**

Brazil

**Hong Dai**

USA

**Jesse Damsker**

USA

**Jaroslaw Dastych**

Poland

**Baishakhi Datta**

Germany

**John De Kruif**

Netherlands

**Giada De Palma**

Canada

**Yves Denizot**

France

**Feici Diao**

USA

**Alvaro Diaz**

Uruguay

**Donglin Ding**

China

**Jose Clovis Do Prado**

Brazil

**Bouchra Edderkaoui**

USA

**Hermann Eibel**

Germany

**Steffen Eisenhardt**

Germany

**Rashika El Ridi**

Egypt

**Kathrin Eller**

Austria

**Klaus Erb**

Germany

**Maria Farcet**

Austria

**Epifanio Fichera**

Italy

**Luigi Franchi**

USA

**Jianing Fu**

USA

**Megumi Funakoshi-Tago**

Japan

**Cem Gabay**

Switzerland

**Awen Gallimore**

USA

**Dong Gao**

USA

**Olivier Garraud**

France

**Howard Gendelman**

USA

**Bernhard F Gibbs**

USA

**Tibor Glant**

USA

**Arturo Gonzalez-Quintela**

Spain

**Steven Goodman**

USA

**Arash Grakoui**

USA

**Bruno Gran**

USA

**Alisa Gruden-Movsesijan**

Serbia

**Tabouret Guillaume**

France

**Sabina Halappanavar**

Canada

**Bruce Hall**

Australia

**Laurie Harrington**

USA

**Catherine Hawrylowicz**

USA

**Zhiheng He**

USA

**Helena Helmby**

USA

**Martin Hewison**

USA

**Masaki Hikida**

Japan

**Keiji Hirota**

Japan

**Matthew Hogan**

Canada

**Elham Hossny**

Egypt

**James Hutchinson**

Germany

**Akihiro Ishizu**

Japan

**Satoshi Ito**

Japan

**Rao Ivatury**

USA

**Roland Jacobs**

Germany

**Brendan Jenkins**

Australia

**Ping Jin**

USA

**Kari Johansen**

Sweden

**Stephen Jolles**

USA

**Liz Jury**

USA

**Nobuhiko Kamada**

USA

**Mitsuo Katano**

Japan

**Bodin Khwannimit**

Thailand

**Jae Kim**

South Korea

**Joanna Kirman**

New Zealand

**Scott Kitchen**

USA

**Oliver Holger Krämer**

Germany

**Susanne Kreuzer**

Germany

**Necil Kutukculer**

Turkey

**Byungsuk Kwon**

South Korea

**Hyung-Joo Kwon**

South Korea

**Vera Leibovici**

Israel

**Vanda Lennon**

USA

**James Li**

Hong Kong

**Xiao-Kang Li**

Japan

**Daniel Libraty**

USA

**Brett Lidbury**

Australia

**David J Lim**

USA

**Chen Lin**

China

**Hao Liu**

USA

**Siqi Liu**

USA

**Andrew Lloyd**

Australia

**Linda Lua**

Australia

**Sanjiv Luther**

Switzerland

**Rudolf Manz**

Germany

**Masanori Matsui**

Japan

**Christian Meisel**

Germany

**Peter Michaely**

USA

**Judith Michels**

France

**Shabir Ahmad Mir**

India

**Sung Moon**

USA

**Arnaud Moris**

France

**Marija Mostarica-Stojkovic**

Serbia

**Yukitoshi Nagahara**

Japan

**Toshihiro Nanki**

Japan

**Maria N Navarro**

Spain

**Cuong Nguyen**

USA

**Christopher Nile**

USA

**Alistair Noble**

USA

**Thomas Nutman**

USA

**Nynne Nyboe Andersen**

Denmark

**Susanne Nylen**

Sweden

**Shinji Oki**

Japan

**Judith Olejnik**

USA

**Darren O'Rielly**

Canada

**Edgar Overton**

USA

**Ferda Ozkinay**

Turkey

**Stephen Pang**

Canada

**Simon Panzer**

Austria

**Stephane Paul**

France

**Ross Paveley**

USA

**Linda Peelen**

Netherlands

**Helene Perrin**

France

**Karlheinz Peter**

Australia

**Tom Petro**

USA

**Paul Pfeffer**

USA

**Eric Pinaud**

France

**Alessandro Poggi**

Italy

**Bojan Polic**

Croatia

**Bruno Pozzetto**

France

**Rupert Quinnell**

USA

**Michael Rajnik**

USA

**Angela Raucci**

Italy

**Pedro Reche**

Spain

**Jay Reddy**

USA

**Sai Reddy**

Switzerland

**Huan Ren**

China

**Blanca Restrepo**

USA

**Joseph Reynolds**

USA

**Yolande Richard**

France

**Eleanor Riley**

USA

**Florence Robert-Gangneux**

France

**Christopher Roman**

USA

**Matthew Rondina**

USA

**Helene Rosenberg**

USA

**Jan Rupp**

Germany

**Buka Samten**

USA

**Ram Savan**

USA

**Mirta Schattner**

Argentina

**Stephan Schlickeiser**

Germany

**Anja Scholzen**

Netherlands

**Alessandro Sette**

USA

**Sadhna Sharma**

India

**Akira Shibuya**

Japan

**Takeshi Shimosato**

Japan

**Yehuda Shoenfeld**

Israel

**Frank Siebenhaar**

Germany

**Joanna Siennicka**

Poland

**Roy Silverstein**

USA

**Udai Singh**

USA

**Christopher Snyder**

USA

**Ana E Sousa**

Portugal

**Sherry Spinelli**

USA

**Mile Stanojcic**

Canada

**Henry Stephens**

USA

**Raymond Steptoe**

Australia

**Hendrik Streeck**

USA

**Kaihong Su**

USA

**Hiroshi Tanaka**

Japan

**Alberto Tedeschi**

Italy

**Julie Tellier**

Australia

**Magali Terme**

France

**Thomas Thornley**

USA

**Jean-Paul Tomasi**

Belgium

**Panagiotis Tourlomousis**

USA

**Marita Troye-Blomberg**

Sweden

**George Tsarianski**

Bulgaria

**Hiroki Tsukamoto**

Japan

**Judith Varner**

USA

**Ramachandran Vignesh**

India

**Eduardo Villablanca**

Sweden

**Peter Villiger**

Switzerland

**Mark Wallet**

USA

**Ruipeng Wang**

USA

**Wilmore Webley**

USA

**Theresa Whiteside**

USA

**Julie Williams**

USA

**Simon Willis**

Australia

**Christer Wingren**

Sweden

**Chack Kie Wong**

Hong Kong

**Grzegorz Woszczek**

USA

**Qinglin Wu**

USA

**Steven Wu**

USA

**Takahiro Yamazaki**

Japan

**Chuen-Mao Yang**

Taiwan

**Sule Yavuz**

Turkey

**Howard Yim**

Australia

**Yangsheng Yu**

USA

**Rana Zabad**

USA

**Nátalli Zanete Pereira**

Brazil

**Mariastella Zannini**

Italy

**Michael Zemlin**

Germany

**Xiaofeng Zheng**

China

**Rafal Zwiech**

Poland

